# Response of spike-wave discharges in aged APP/PS1 Alzheimer model mice to antiepileptic, metabolic and cholinergic drugs

**DOI:** 10.1038/s41598-020-68845-y

**Published:** 2020-07-16

**Authors:** Nanxiang Jin, Sofya Ziyatdinova, Irina Gureviciene, Heikki Tanila

**Affiliations:** 0000 0001 0726 2490 grid.9668.1 A. I. Virtanen Institute, University of Eastern Finland, PO Box 1627, 70211 Kuopio, Finland

**Keywords:** Neuroscience, Biomarkers

## Abstract

Epileptic nonconvulsive spike-wave discharges (SWDs) are commonly seen in amyloid plaque bearing transgenic mice but only rarely in their wild-type littermates. To shed light on their possible treatment options, we assessed the effect of drugs with variable and known mechanisms of action on the occurrence of SWDs in aged APPswe/PS1dE9 mice. The treatments included prototypic antiepileptic drugs (ethosuximide and levetiracetam), donepezil as the typical Alzheimer drug and atropine as an antagonistic effect, GABA_B_ antagonist CGP-35348, and alternate energy substrates beta-hydroxybutyrate (BHB), pyruvate and lactate on the occurrence of SWDs in aged APPswe/PS1dE9 mice. All agents were administered by single intraperitoneal injections at doses earlier documented to be effective and response was assessed by recording 3 h of video-EEG. Atropine at 25 mg/kg significantly decreased SWD occurrence in all behavioral states, and also resulted in altered frequency composition of SWDs and general EEG slowing during sleep. Ethosuximide at 200 mg/kg and levetiracetam at 75 mg/kg effectively suppressed SWDs only during a period of mixed behavioral states, but levetiracetam also increased SWDs in sleep. BHB at 1 g/kg decreased SWDs in sleep, while both pyruvate and lactate at the same dose tended to increase SWD number and total duration. Unexpectantly, donepezil at 0.3 mg/kg CGP-35348 at 100 mg/kg had no effect on SWDs. These findings call for re-evaluation of some prevailing theories on neural circuit alternations that underlie SWD generation and show the utility of APP/PS1 mice for testing potential new treatments for nonconvulsive epileptic activity related to Alzheimer pathology.

## Introduction

Elderly individuals with Alzheimer’s disease (AD) have about eightfold risk of epileptic seizures compared to age-matched population^1^. Moreover, over 40% AD patients with no history of epilepsy show subclinical epileptiform activity in EEG/MEG recordings, which unfavorably influences the disease process^2^. This observation raises the question whether this kind of ‘silent’ epileptic activity needs to be medically treated. However, drug treatment for epileptic activity in AD patients is challenging due to known cognition impairing effects of most anti-epileptic drugs^3^. In addition, according to a recent large register study use of anti-epileptic drugs (AEDs) with known cognitive side effects may increase dementia risk in elderly individuals^4^. Notably, for most patients in this register study AEDs were prescribed as mood stabilizers for behavioral symptoms, not for detected epileptic activity. So far, no published study has assessed the potential benefit vs. side effects of AEDs or other medication for ‘silent’ epileptic activity in AD or mild cognitive impairment (MCI) patients. However, there are several ongoing phase 2 clinical trials on cognitive effects of levetiracetam on AD or MCI patients, some of which also include EEG for epileptic activity (NCT04004702, NCT03489044, NCT03875638 at https://clinicaltrials.gov/ct2/show/). The selection of levetiracetam among all AEDs was based on its beneficial cognitive effects in a small previous study on MCI patients^5^, as well as its ability to improve memory and reduce epileptic spiking in APP transgenic mice^6^. However, as long as the mechanism of AD-related epileptic activity is not known, we do not know whether established AED regimes can be applied to MCI/AD patients with ‘silent’ epileptic activity or whether other established drugs with a completely different mechanisms would prove equally effective.

We have recently reported frequent occurrence of spike-wave discharges (SWDs) that are not associated with muscle activity in widely used amyloid plaque producing transgenic APPswe/PS1dE9 mice, while SWDs were seldom seen in their wild-type littermates^7^. SWDs have been previously linked with absence seizures in genetic rat models of absence epilepsy^8,9^ and may provide a surrogate marker for testing the effects of various pharmaceutical treatments for nonconvulsive epileptic activity in AD patients, although an increase in SWDs has not been reported in AD patients. In the only preclinical drug study to date, Nygaard and colleagues^10^ assessed the responses of two common AD mouse models (APP/PS1 and 3xTg-AD) with brain amyloidosis to four AEDs, brivaracetam, ethosuximide, levetiracetam and phenytoin. Of these, ethosuximide, and to a lower extent, brivaracetam and levetiracetam were able to significantly reduce SWDs in both mouse lines, whereas phenytoin was ineffective. This pioneer study indicates that SWDs are pharmaceutically treatable and shows their potential for preclinical screening of new treatment agents against nonconvulsive epileptic discharges in MCI/AD.

Using SWDs as a model, the present study set out to determine the effect of three types of potential treatments against epileptic spiking without visible manifestations: (1) conventional AEDs, (2) ACh and GABA modulating drugs, and (3) metabolic compounds. As AEDs we selected ethosuximide since it is the prototype drug effective for absence seizures, which are associated with SWDs in genetic rat models of absence epilepsy, and levetiracetam that has little reported cognitive side effects^3^ and has proven effective against spiking activity in APP transgenic mice^6^. Reduced tone in basal forebrain cholinergic neurons may contribute to SWDs, as lesion of the cholinergic basal nucleus induces SWDs in rats^11^. Therefore, we selected donepezil as the prototypic acetyl cholinesterase inhibitor, and wanted to test also whether atropine as muscarinic receptor antagonist would exert an opposite effect. Imbalance between fast GABA_A_ and slow GABA_B_ inhibition was found in thalamus in a genetic rat model of absence epilepsy and suggested to be one underlying mechanism of SWDs^12^. Therefore, we aimed to decrease GABA_B_ tone in our APP/PS1 mice with a selective GABA_B_ antagonist CGP-35348, which reduced occurrence of SWDs in an absence seizure rat model^13^. Finally, ketogenic diet has been proven to be effective in many childhood epilepsies, including absence epilepsy^14^. Furthermore, our previous data suggest that chronic administration of the key ketone substance beta-hydroxybutyrate (BHB) in combination with pyruvate reduces the occurrence of epileptiform discharges (largely overlapping with SWDs) in APP/PS1 mice^15^. Recently, BHB has been shown to specifically bind to hydroxycarbolic acid (HCA) 2 receptor in the brain^16^, suggesting that it may exert its effect on brain excitability in a receptor-mediated action in addition to its metabolic action. Systemically administered pyruvate converts rapidly to lactate in the periphery but also reaches measurable levels in the brain when administered at large doses^17,18^. There is recent evidence that also lactate binds specifically to its own receptor, HCA 1, in the brain, and may influence neuronal excitability through this mechanism besides acting as an energy substrate^19^. We hypothesized that a single injection of BHB, pyruvate or lactate would reveal the putative receptor mediated action of these substances on neuronal excitability more specifically than chronic administration.

This study focused on treatment options of SWDs in a relevant mouse model of AD, aged APPswe/PS1dE9 mice with fully developed amyloid plaque pathology^20^, impaired cholinergic neurotransmission^21^, and robust memory impairment^22^. The emphasis of the study was to compare drugs with different known mechanism of actions rather than finding the optimal dose for each drug. Therefore, we chose the highest dose with no documented motor side effects. All drug responses were studied under different behavioral states (sleep, waking immobility, movement and a mixed state). The behavioral state proved to be an important determinant of the SWD responses to treatment, since all significant drug effects, with the exception of atropine, were seen only in a certain state.

## Materials and methods

### Animals

Eleven 17- to 18-month-old male APPswe/PS1dE9 (APP/PS1) mice^23^ were randomly selected from the colony at University of Eastern Finland based on founders from Johns Hopkins University, Baltimore, MD, USA (by D. Borchelt and J. Jankowsky). The mice were backcrossed to C57BL/6J strain for 21 generations. The mice were housed in a controlled environment (temperature 22 ± 1 °C, humidity 50–60%, lights on 07:00–19:00) with food and water available ad libitum. All animal procedures were carried out in accordance with the guidelines of the European Community Council Directives 86/609/EEC and approved by the Animal Experiment Board of Finland.

### Electrode implantation

The electrode implantation has been described earlier^7^. Briefly, under isoflurane anesthesia (4.5% for induction, ~ 2% for maintenance) two cortical screw electrodes were attached symmetrically to the left and right frontal bone (AP+ 1.7, ML+ and – 1.8). Further, two screws fixed on the occipital bone served as the ground and common reference. All screws served also as anchors for dental acrylic cement in which a miniature connector (Mill-Max, NY, USA) was imbedded. Additionally, a stainless-steel wire electrode (Formvar insulated, diameter 50 mm, California Fine Wire Company Co, Grover Garcia, CA, USA) was inserted between the neck muscles for electromyogram (EMG) recording. After surgery, the mouse received carprofen (5 mg/kg, i.p., Rimadyl, Vericore, Dundee, UK) daily for postoperative analgesia for up to 3 days, and antibiotic powder (bacitracin 250 IU/g and neomycinsulfate 5 mg/g, Bacibact, Orion Finland) was applied on the wound.

### Drug administration

Eight compounds, ethosuximide, levetiracetam, donepezil, atropine, GABA_B_ antagonist CGP 3,534, beta-hydroxybutyrate (BHB), pyruvate, and lactate (details in Table 1) intermingled with two saline controls were administered intraperitoneally (i.p.) to each animal in a random order. To compare several treatments with difference mechanism of action, we had to limit to a single dose of each compound. The selection of doses was based on the available literature. Ethosuximide (ESM) at the dose 200 mg/kg and levetiracetam (LEV) at the dose of 75 mg/kg were estimated to be effective but nonsedative^6^. Donepezil (DPZ) at 0.3 mg/kg but no more at 0.6 mg/kg has improved learning and memory in APP transgenic mice^24^, while atropine (ATR) at 50 mg/kg has been the standard way to suppress hippocampal theta in rats and mice^25^. However, this dose resulted in hyperactivity in our pilot experiments, and therefore, we needed to reduce the dose to a half. The GABA_B_ antagonist CGP-35348 reduced occurrence of SWDs in an absence seizure rat model at a dose of 100 mg/kg^13^, which was selected for the present study. BHB, lactate (LAC) and pyruvate (PYR) have been shown to be approximately equipotent in increasing brain levels of glucose as alternate energy supplies^18^. The dose of 1 g/kg of pyruvate or lactate has been shown to result in sustained increase brain levels of these substances^17^. The preparation and injection of drug solutions were double-blinded. Injections were given at the same time of day 30 min before video-EEG recordings started. The washout between two subsequent drugs was 1 week.

**Table 1:** Details of tested drugs and the number of animals in which the effect was tested.

Drug (abbreviation)	Animal number	Vehicle	Function	Dose (i.p.)	Volume	Source
Ethosuximide (ESM)	8	0.9% NaCl	Absence seizure drug	200 mg/kg	0.1 ml/10 g	Sigma Aldrich
Levetiracetam (LEV)	9	0.9% NaCl	Anticonvulsant	75 mg/kg	0.1 ml/10 g	Carbosynth, Comptom, UK
Donepezil-hydorchloride (DPZ)	9	0.9% NaCl	AChE inhibitor (AD drug)	0.3 mg/kg	0.1 ml/10 g	Sigma Aldrich
Atropine (ATR)	8	0.9% NaCl	mAChR antagonist	25 mg/kg	0.1 ml/10 g	OrionPharma, Turku, Finland
CGP 35348 (CGP)	9	0.9% NaCl	GABA(B) antagonist	100 mg/kg	0.1 ml/10 g	Abcam
Na-Pyruvate (PYR)	9	dH2O	Energy substrate	1.0 g/kg	0.1 ml/10 g	Sigma Aldrich
Na-Beta-hydroxybutyrate (BHB)	9	dH2O	Energy substrate, ketogenic diet, HCA2 agonist	1.0 g/kg	0.1 ml/10 g	Sigma Aldrich
Na-lactate (LAC)	9	dH2O	Energy substrate, HCAR1 agonist	1.0 g/kg	0.1 ml/10 g	Sigma Aldrich
0.9% NaCl (Saline)	9		Vehicle		0.1 ml/10 g	

### Video-EEG acquisition

After a week of recovery from the surgery, the mice were first familiarized with the recording environment for 2 days. The video-EEG recording took place in a circular frame made of brown compressed paper (diameter 18.5 cm, wall height 18 cm) located on a translucent glass plate, which was illuminated from below with multiple white LEDs. Two circular frames were placed on the same glass plate and two mice were recorded simultaneously. One end of recording wire was attached to a unity-gain preamplifier (Plexon HST/16o50-G1-R11, Dallas, USA) that connected to the imbedded connector (Mill-Max, NY, USA) on the mouse’s head, while the other end of recording wire was connected to an AC amplifier (A-M Systems 3600, Sequim, WA, USA; gain 1,000, analogue band-pass 1–1,000 Hz). The amplified signal was digitized at 2 kHz per channel (DT2821series A/D board; Data Translation, Marlboro, MA, USA). The behavior of the animals was recorded ~ 20 fps using a webcam (Live! Cam, Video IM Pro, Creative, Dublin, Ireland) that was mounted over the arena. Synchronized electrophysiological signals and behavioral videos were acquired and saved in a computer with Sciworks 5.0 program (DataWave Technologies, Loveland, CO, USA). Each mouse underwent one recording session of 3 h per drug either in the morning or afternoon, but always within light phase of the day (lights on 7:00 to 19:00).

### Assessment of behavioral states

The videos were analyzed offline with Ethovision software (version XT, Noldus Information Technology bv, Wageningen, the Netherlands). Based on the tracked mouse coordinates in every video frame, we smoothened the trajectory and corrected unrealistic mouse locations with customized Matlab programs. First, we removed unrealistically sharp turns. Every two subsequent coordinates make a trajectory vector. If the angle of the vector was bigger than 135° or smaller than − 135 (sharp turning back), the second coordinate was replaced by the mean of the 2nd and 3rd points. Second, we corrected star-shaped and non-mouse object tracking by comparing the distance from one point to subsequent 4 points. The nearest point of the four was taken as the realistic location, and then the correction continued from the next “nearest point”. Third, we smoothened the overall trajectory with the Matlab function SMOOTHN using the default parameters^26^.

Next, we assigned each 10-s sweep to one of the four behavioral states: sleep, waking immobility, movement, and mixed states. First, we measured instant speeds between every two neighboring mouse locations the in video based on the distance shift and the lapse of time between them. If the instant speed was higher than 0.5 cm/s this video frame was assigned as movement, otherwise immobility. Second, if a single frame with movement was preceded and followed by 10 frames of immobility, this frame was also assigned as immobility. Third, in any immobility period that was longer than 30 s, all video frames from the 31st second till the end of this immobility period were assigned as sleep. Fourth, every 10 s was arbitrarily cut as a sweep. Within each sweep, if the number of frames in any state exceeded 60% of the total frame number, that sweep was assigned the same as the dominant state; if no single state exceeded 60%, this sweep was marked as ‘mixed state’. Five types of transitions can take place during the mixed state: (1) moving→waking immobility, (2) waking immobility→moving, (3) waking immobility→sleep, (4) sleep→waking immobility (with a brief movement epoch in between) and (5) sleep→moving. We did not analyze each type of transition separately as that would yield too few cases per mouse for statistics. Therefore, we decided to pool all transitions into one behavioral state. The categorization of SWDs into sleep, immobility, movement or mixed state was based solely on this video tracking to avoid misinterpreting direct drug effects on EEG as a change in the behavioral state.

### EEG data analysis

All signals were normalized to amplification and analyzed offline in Matlab (Mathworks, Natick, MA, USA; R2018b). First, every channel in each recording was divided into 10-s sweeps to facilitate artifact exclusion, synchronization with behavioral state assignment from video, and further analysis. Occasional artifacts (e.g., bad contact, jerky movements, etc.) were estimated by calculating the power spectrum distribution in each sweep; when the summed power from 1 to 100 Hz was higher than 2 × 10^4^ mV^2^/Hz, this sweep was excluded from any further analysis. Besides, we visually checked recording quality and cut out noisy sweeps manually from individual channels. All channels that had fewer than 60 noisy sweeps (= 10 min) were qualified into the database.

**Figure 1:**
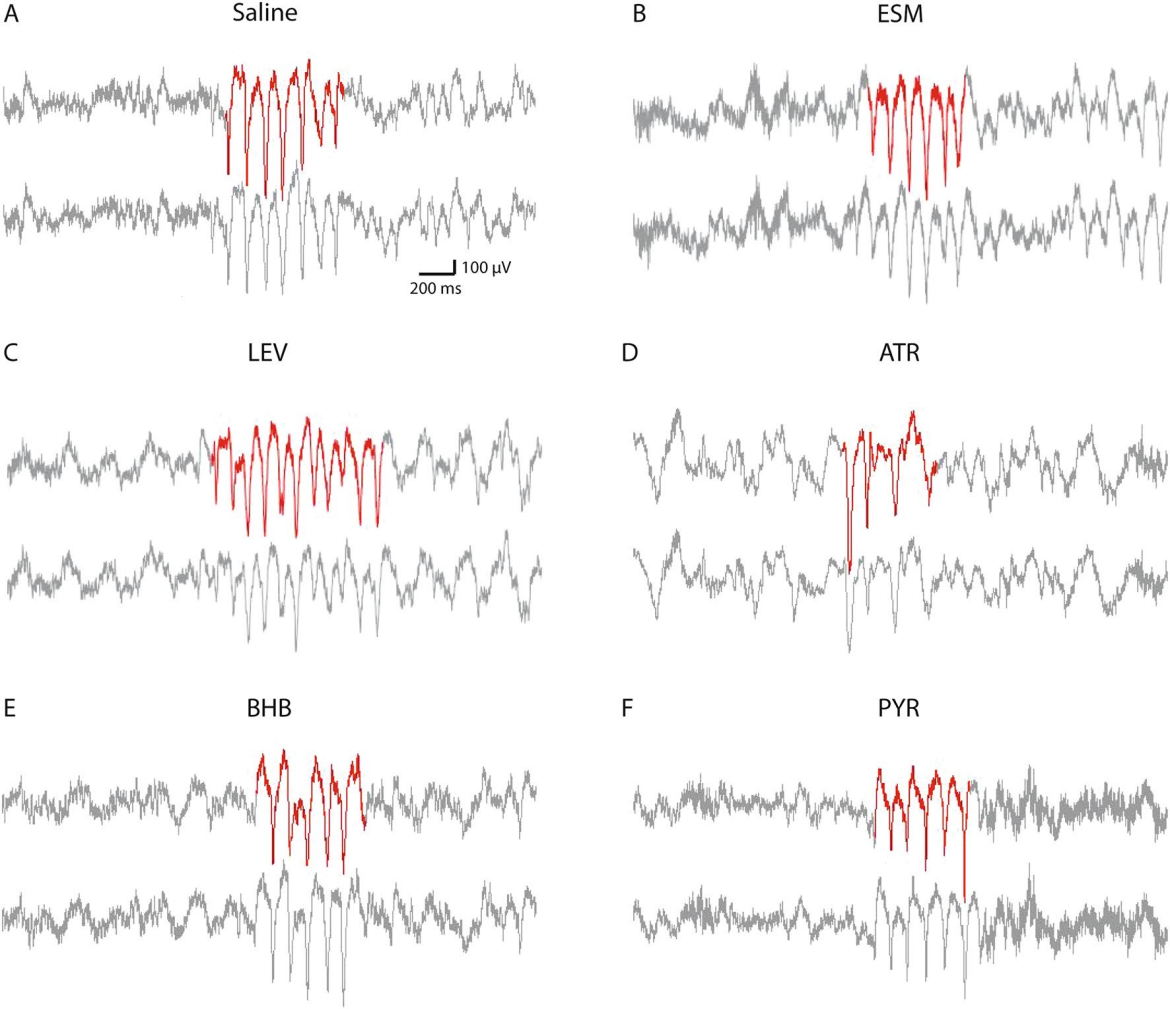
Spike-wave discharge (SWD) examples in EEG recordings under different drug treatments. Upper channels from right and lower channels from left frontal cortex. SWDs in the analyzed right frontal cortical channel are marked in red color. Scale bar is shown in the figure. *ESM* ethosuximide, *LEV* levetiracetam, *ATR* atropine, *BHB* beta-hydroxybutyrate, *PYR* pyruvate.

Two types of SWDs were reported in WAG/Rij rats, one type bilateral over the entire cortex and the other unilateral restricted to the parieto-occipital cortex^27^. However, we could identify only the first type of SWDs with closely similar pattern in both hemispheres (Fig. 1). Therefore, only the EEG channel from the right hemisphere of each mouse was analyzed. SWDs were screened out from EEG with visual inspection by two experienced colleagues. To increase screening efficiency, we used a customized Matlab software to prescreen all SWD candidates (including all real SWDs and many fake positive SWDs) out from the EEG as previously reported^7^. In brief, we band-pass filtered the raw EEG signal between 7 and 23 Hz, and then calculated an envelope by taking the absolute values of Hilbert-transformed filtered signal. Next, a positive threshold was set above the envelope mean. The two crossing points between the envelope and the threshold marked the two ends of a candidate SWD event. Only the events that were longer than 400 ms or had more than three spike-wave cycles were included in the SWD collection. The threshold was carefully adjusted by visual inspection per record file until no true events were excluded and as few false events as possible included. Between individual mice, this threshold varied between 1.8 and 3.4 SD in the saline recordings. Finally, we used a customized user interface to help two experienced human raters blinded to the treatment to decide the acceptance versus rejection on each candidate event based on the regularity of the spike-wave pattern. In each mouse, the number and summed duration of true SWD events that occurred in each behavioral state were both normalized by the total hours in that state because the occurrence of SWDs is highly behavior state-dependent^28^. The SWD number, total duration, mean duration of a single event and animal moving time were first assessed by ANOVA for repeated measures (ANOVA-RM) using IBM SPSS Statistics (version 25) to test for overall difference between the treatments. If significant, we further applied paired t-test to evaluate the difference between saline and each drug treatment. Type I error from multiple comparisons was corrected with the false discovery rate (FDR) correction^29^. Only the first saline session was used as the control in paired t-test. Further, when two treatments included different number of animals, only overlapping ones were included in the paired t-test. Because SWD seldom happens during moving, we did not run statistics of SWDs during the identified moving states. The power spectral density (PSD) of all SWD events in each drug group was estimated as follows. All SWDs on the same drug during immobility were picked out and merged, and then Welch’s averaged modified periodogram method of spectral estimation (default MATLAB parameters) was applied to calculate PSD of the merged time series based on 10-s sweeps. The absolute PSD of the total EEG was calculated in the same way, and then averaged for each behavioral state and drug. Similarly, the relative PSD of the total EEG was pre-processed so that PSD of every 1-Hz bin was divided by summed PSD across 1–30 Hz in each mouse and then multiplied 5,000 times for better visualization. To compare the PSD between saline and each drug treatment group, we ran multiple t-tests for each 1-Hz bin and applied FDR correction across 1–30 Hz for multiple comparisons. The statistical threshold was set at p < 0.05. For all PSD related analyses, the control group was composed of pooled data from two saline sessions.

## Results

### Only ATR altered locomotor activity of APP/PS1 mice

**Figure 2:**
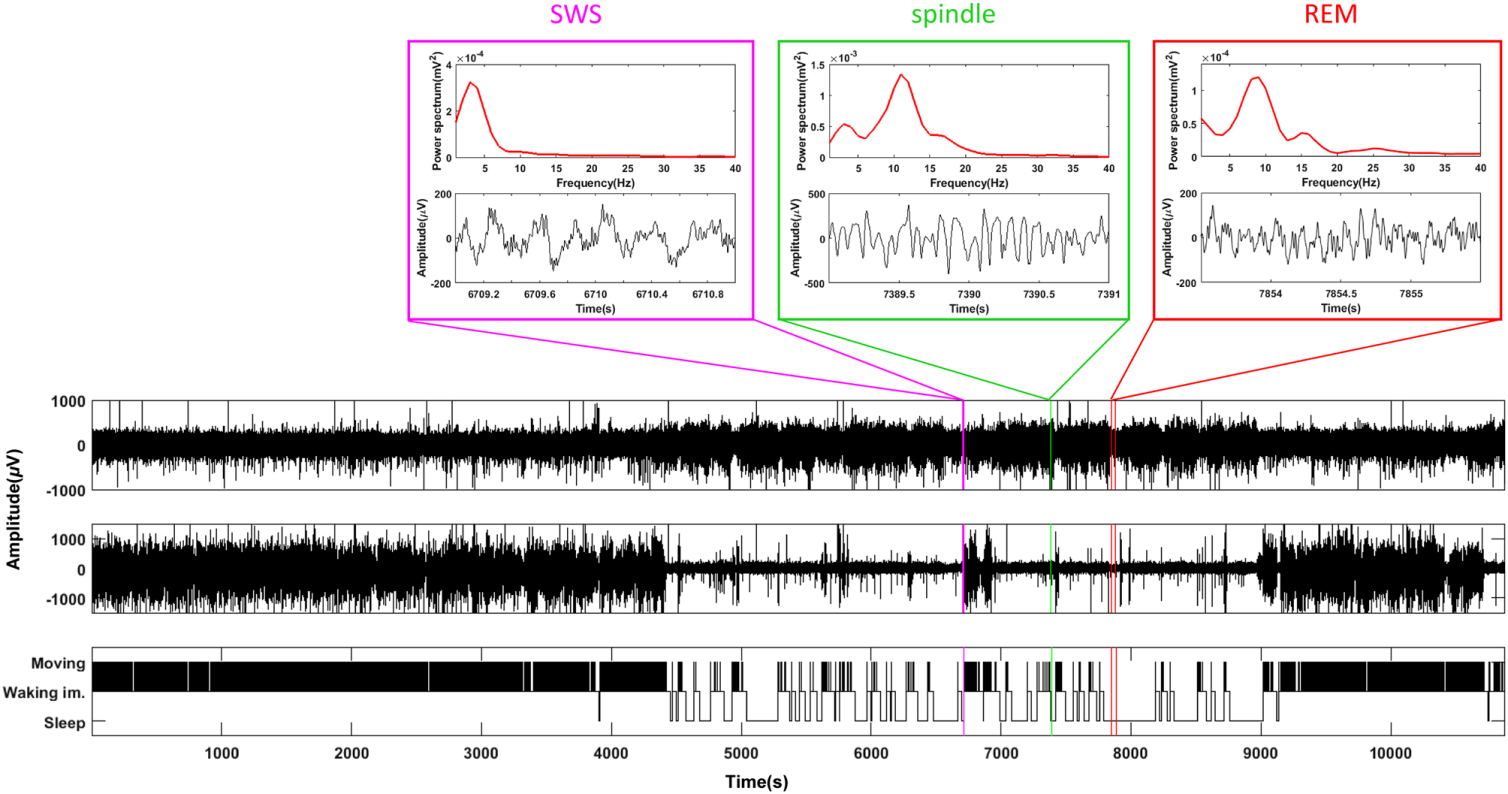
Typical pattern of behavioral stages during the recording session. Lower panel: Recordings of a mouse with saline injection during the entire 3 h session. Channels from top to bottom: cortical EEG, EMG, video staging (Moving, Waking immobility and Sleep). Upper panels: 2-s examples of raw EEG and power spectral density of slow-wave sleep (SWS), superficial sleep with a sleep spindle and REM sleep.

**Figure 3:**
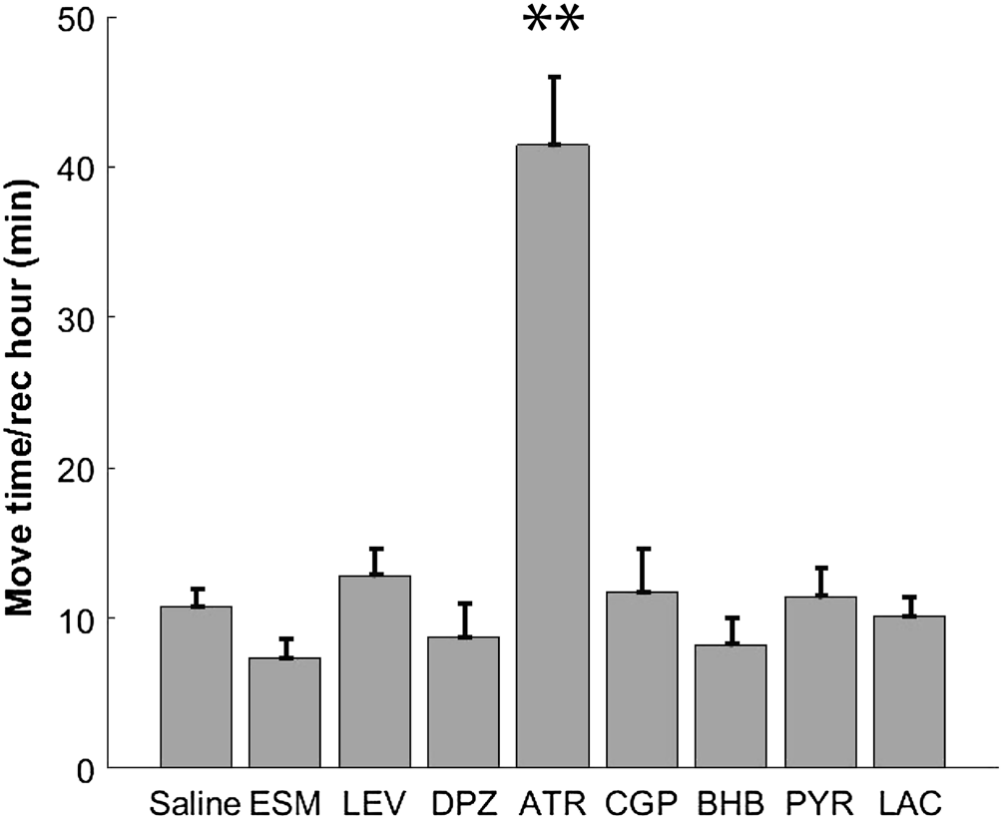
Locomotor activity with drug administration. ATR increased the moving time per recording hour while other drugs did not change locomotor activity of the mice. **p < 0.01 in paired t-test with false discovery rate (FDR) correction.

In general, the mice first spent about half an hour in each recording session to explore the arena, and then shifted to immobility-exploration alternation. The last hour of each session the mice mostly spent in sleep, including short bouts of slow-wave sleep and REM but mostly superficial sleep characterized by sleep spindles. Figure 2 illustrates a typical pattern of behavioral stages during a 3-h recording session. Mice under ATR treatment were moving in almost 2/3 of the recording time on average, while under other drugs the mice spent about 1/6 of the recording time in moving (Fig. 3). ANOVA-RM showed an overall difference in the mean moving time between the treatments (F_2.8/16.7_ = 22.06, p < 0.001), while only ATR significantly increased locomotor activity (i.e. decreased total immobility duration) of the mice in post-hoc FDR corrected paired t-test.

### SWD responses to acute drug administration were behavioral state-dependent

**Figure 4:**
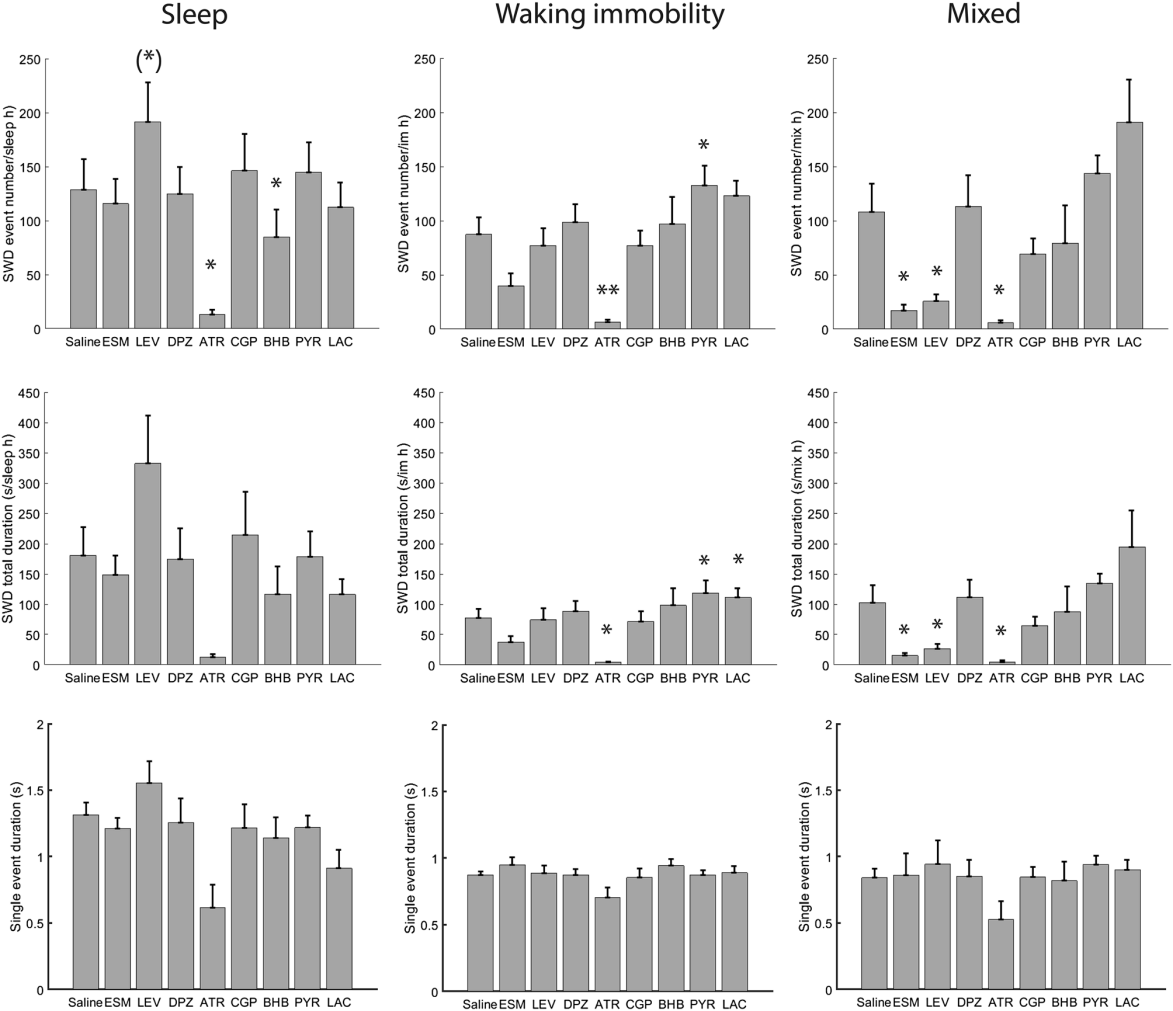
Drug responses of SWDs in APP/PS1 mice under sleep, waking immobility and mixed states. Except single SWD duration in mixed state, all other parameters showed significant difference in ANOVA with repeated measures. Further paired t-test uncovered that ATR decreased SWD event number and total duration in all behavioral states. ESM decreased event number and total duration only in the mixed state, while BHB specifically decreased SWD number in sleep. In contrast, PYR significantly increased the SWD number and total duration during waking immobility. Interestingly, LEV displayed bidirectional effects, in that it increased SWD number during sleep and reduced SWD number and total SWD duration during mixed state. (*)p = 0.05, *p < 0.05, **p < 0.01 in paired-samples t-test with FDR correction^29^. Error bars show group means + SEMs. Animal numbers and abbreviations for drugs are listed in Table 1.

Typical SWDs under the influence of the studied drugs are illustrated in Fig. 1. We evaluated the effect of the studied drugs on the generation of SWDs during 3 h of EEG recording by counting the SWD number, total SWD duration per sleep/immobility/mixed state hour, and the mean duration of a single SWD event (Fig. 4). Firstly, no statistically significant difference in any measured parameter was found between two saline sessions after intervening drug treatments (all p values > 0.05 in paired t-test), so we used only the first saline session in the comparison with other drug treatments. Secondly, except for a single SWD duration in the mixed state, all other test parameters showed significant differences between the treatments in an ANOVA for repeated measures (ANOVA-RM) after a Greenhouse–Geisser correction (1) SWD number: sleep F_3.4/20.7_ = 6.9, p = 0.002; immobility F_3.2/19.2_ = 11.4, p < 0.001; mixed F_2.1/12.5_ = 7.7, p = 0.006; (2) total SWD duration: sleep F_2.8/16.7_ = 4.6, p = 0.017; immobility F_2.5/15.0_ = 8.5, p = 0.002; mixed F_1.5/8.7_ = 5.4, p = 0.037; (3) single event duration: sleep F_2.6/15.4_ = 4.4, p = 0.023; immobility F_2.2/13.2_ = 4.3, p = 0.033; mixed F_3.5/21.1_ = 1.1, p = 0.38). Following post-hoc t-tests showed that compared to saline administration ATR significantly decreased SWD occurrence in all three behavioral states (sleep, waking immobility and mixed), but also resulted in EEG slowing (Figs. 6, 7), whereas donepezil had no significant effects. Furthermore, ESM effectively suppressed SWDs only during the mixed state and decreased both absolute and relative EEG power at ~ 8–10 Hz (peak power frequency of SWD), while BHB decreased the SWD number specifically in sleep and decreased relative PSD from 10 to 14 Hz (near SWD peak frequency). On the other hand, PYR at the same dose significantly increased SWD number and total duration during waking immobility, while LAC at the same dose had no significant effects. Interestingly, LEV increased SWD occurrence during sleep but decreased the SWD number in the mixed state. Unexpectantly, CGP at a dose that was effective in a rat model^13^ had no significant effects. None of the drugs had a significant effect on the duration of a single SWD (Fig. 4, bottom row).

### ATR changed the dominant frequency of individual SWDs

**Figure 5:**
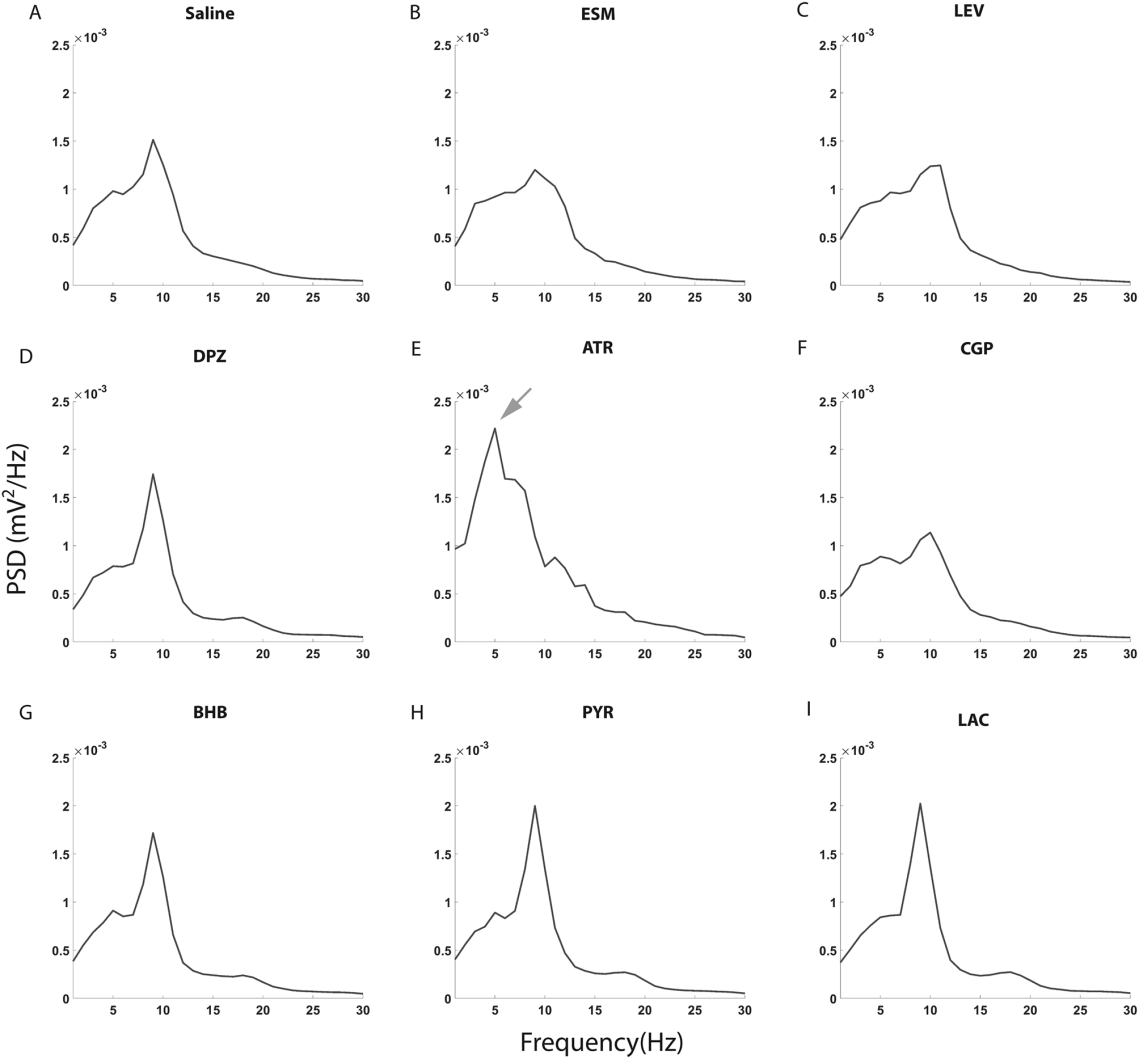
PSD of merged SWDs with saline and drug treatments. (**A**) The PSD of SWD shows a characteristic peak at 9 Hz under saline sessions. The power distribution in ESM (**B**), LEV (**C**) and CGP (**F**) sessions display a decreased peak amplitude and slight frequency shift to 10 Hz. (**E**) ATR induced a dramatic shift of the SWD power peak to 5 Hz (grey arrow), while the remaining treatments (**D**, **G**, **H**, **I**) did not differ from the saline session.

We further analyzed the spectral composition of individual SWDs under dosing of all drugs (Fig. 5). The SWDs occurred approximately to the same extent in sleep, awake immobility and mixed states under saline (Fig. 4) but very rarely during movement. To collect a sufficient number of events, we pooled all SWDs during sleep, awake immobility and mixed state for the analysis. Compared with saline, SWDs under ATR showed a highly distorted PSD, with dramatically increased power at 1–8, 13–14 Hz and a new peak at 5 Hz (grey arrow) (Figs. 1D, 5E). Whereas ESM decreased peak power at 9 Hz (Figs. 1B, 5B), DPZ, BHB, PYR and LAC enhanced the power peak at 9 Hz (Figs. 1E,F, 5D,G–I). LEV and CGP in turn decreased the peak and slightly shifted it to 10 Hz (Figs. 1C, 5C,F).

### ATR increased overall EEG delta frequency during sleep and delta-beta frequency during immobility, moving and mixed state

**Figure 6:**
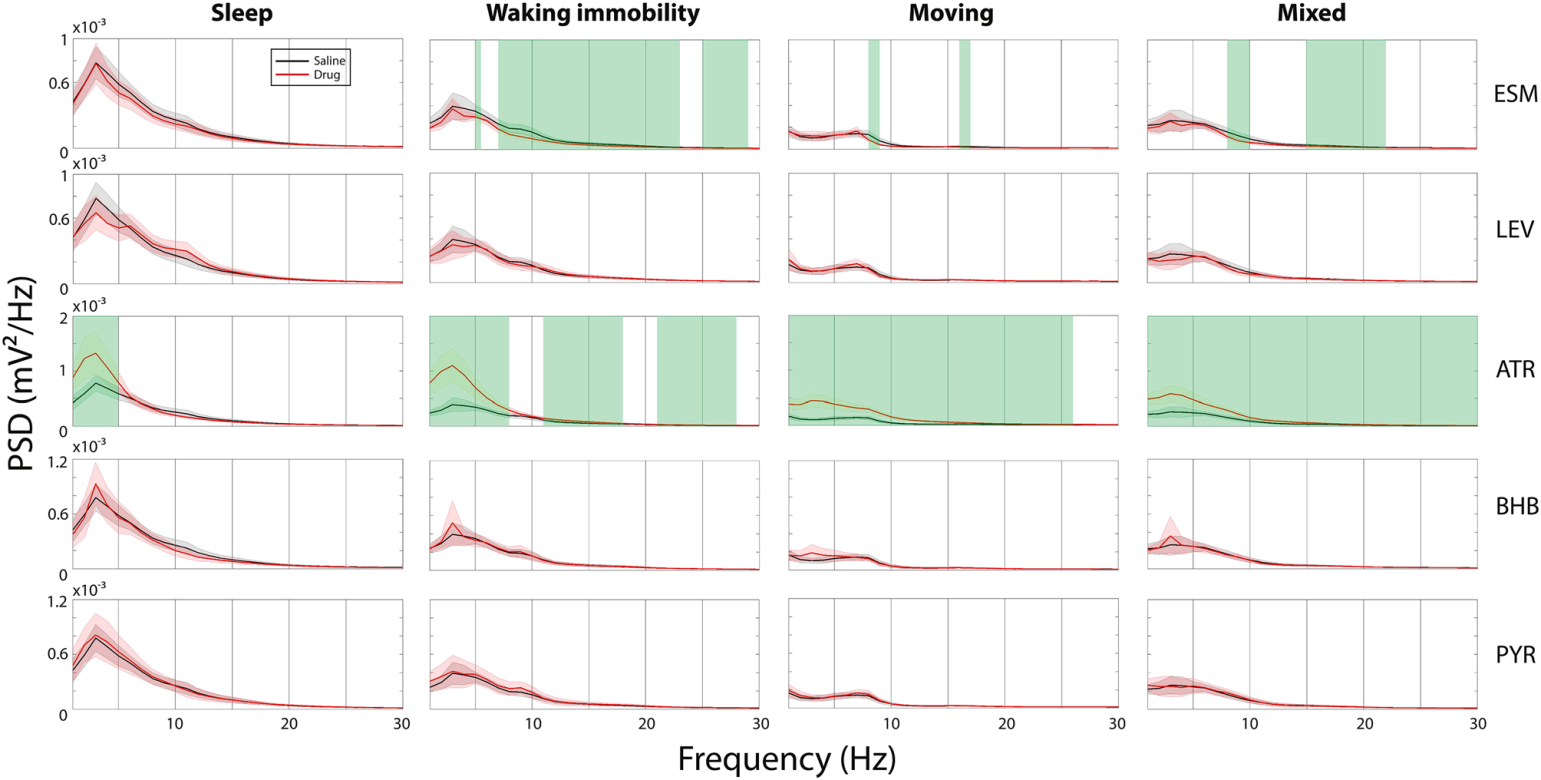
Comparison of absolute mean PSD (± SD) within 1–30 Hz between effective drug administration and saline sessions in different behavioral states. ATR increased delta-low theta (1–5 Hz) power in sleep and delta-beta (1–25 Hz) power in other states. ESM decreased delta power (1–2 Hz) and theta-beta power (7–20 Hz) during waking immobility and reduced SWD ‘resonance’ power (8–11 Hz) in movement and mixed states. Other drugs did not change EEG power distribution in any state. Green shading denotes frequency ranges where the drug session differed from the saline session at 0.05 level in FDR corrected independent samples t-test. All plots were binned by 1 Hz. For animal numbers see Table 1.

Next, we examined whether the drug treatments with a significant effect on the SWDs altered the overall PSD of the EEG recordings in different behavioral states according to the method we have described in our previous work^7,30^. To confirm that the EEG recovered from drug treatment after a 1-week washout, we first compared PSDs between two saline sessions, and found no difference in any frequency in the examined 1–30 Hz range. Then, we compared absolute and relative PSD of each drug session with the saline session and found that in terms of absolute PSD ATR recordings showed significantly higher power than saline recordings at delta frequency band during sleep and at all frequency bands between 1 and 30 Hz during immobility and mixed states; ESM decreased power at SWD ’resonance’ frequency (9–11 Hz) during waking immobility and mixed states. In contrast, LEV, BHB and PYR did not change the absolute PSD compared to saline recordings (Fig. 6). In terms of relative PSD, ATR significantly reduced power at the SWD ‘resonance’ frequency in all states, whereas ESM decreased such power only during the mixed state and BHB only during sleep. In contrast, LEV increased the relative power between 8 and 13 Hz during sleep, which is in line with increased SWD number during sleep in LEV sessions (Fig. 4).

## Discussion

This study assessed the potential of three categories of agents with known but different mechanisms of action, (1) conventional AEDs, (2) ACh and GABA modulating drugs, and (3) metabolic compounds, to suppress the occurrence of SWDs in aged APP/PS1 mice with fully developed amyloid plaque pathology. The aim was not only to screen for potential individual drugs but also to help understand the underlying mechanisms of SWD generation and inhibition. All agents selected for this study had a ‘track record’ to be potentially effective. Hardly any SWDs were detected during movement, while their number was around 100 per hour during saline sessions in sleep, waking immobility and mixed states (Fig. 4). However, only atropine (ATR) proved effective in suppressing SWDs independent of the behavioral state, while levetiracetam (LEV), ethosuximide (ESM) and beta-hydroxybutyrate (BHB) significantly decreased SWD occurrence only in some behavioral states. This state-dependency was most striking for LEV that decreased the number of SWDs during the mixed state, had no effect during awake immobility and even increased their number in sleep.

Certain rat strains (Genetic Absence Epilepsy Rats from Strasbourg and Wistar-Albino-Glaxo from Rijswijk) generated by selective breeding for generations are characterized by frequent spontaneous SWDs and have been considered genetic models of human absence epilepsy despite two notable differences from the human condition. First, absence epilepsy is typically a childhood epilepsy but SWDs reach their peak occurrence in the rat models only in the adulthood. Second, the dominant frequency of SWDs in the rat models is 7–9 Hz, but around 3 Hz in humans^8^. The strongest argument for validating rodent SWDs as a model of human absence seizures is the similar response to AEDs of these conditions. Ethosuximide is a drug-of-choice for human absence seizure while AEDs effective for generalized seizures, including levetiracetam, show only modest effect at best^31^. In partial agreement with the clinical experience, we found ethosuximide effective against SWDs in aged APP/PS1 mice during the mixed state but being neutral in sleep and waking immobility. On the other hand, levetiracetam that has worked best of all tested drug candidates against single epileptic spikes in APP transgenic mice^6^ was also effective against SWDs during the mixed state, but even increased SWD occurrence during sleep. Our finding appears largely consistent also with an earlier study by Nygaard and coworkers on the same APPswe/PS1dE9 mouse, showing a response to ethosuximide at the same dose and to levetiracetam at a slightly higher dose (75 mg/kg vs. 20 mg/kg); however, a back-to-back comparison is made difficult by less clearly defined behavioral state during recordings in the Nygaard et al. study^10^. Notably, although both LEV and ESM reduced the number of SWDs in the mixed state, they had different effects on the EEG power spectrum during this state. ESM decreased both absolute and relative power at the SWD dominant frequency around 9 Hz while LEV had no significant effects. On the other hand, LEV even increased the relative power of the SWD dominant frequency during sleep, which may explain the observed slight increase in SWD number during sleep in LEV sessions.

It should be further noted that sleep is the most challenging brain state to reliably detect SWDs due to the presence of sleep spindles. Sleep spindles are generated in the same thalamocortical circuit and oscillators as SWDs and only slightly differ in their frequency^32^. When sleep spindles are accompanied by spiking their appearance can be indistinguishable from SWDs on the skull EEG. Therefore, the drug effects on SWD occurrence during sleep should be interpreted with caution. The differential effects on PSD by LEV and ESM are consistent with their known mechanisms of action. LEV inhibits presynaptic Ca^2+^ channels thereby reducing glutamate release^33^, which is expected to generally reduce neuronal excitability but not their oscillations at a specific frequency. On the other hand, ESM reduces T-type Ca^2+^ currents in thalamic neurons^34^ , which is expected to alter the thalamo-cortical spindle oscillations.

The underlying circuitries and neurotransmission of SWDs have been intensively studied over the past three decades, and a current consensus is that the somatosensory cortex, VP, Po, and RT thalamic nuclei are intimately involved in these oscillations^35^. Furthermore, an imbalance between fast GABA_A_ and slow GABA_B_ inhibition was found in thalamus in a genetic rat model of absence epilepsy and suggested to be one underlying mechanism of SWDs^12^. Somatosensory cortex shows the earliest amyloid accumulation in APP/PS1 mice^20^ and thus appears as the most likely site of generation of SWDs in these mice. Reduced GABA_A_ mediated inhibition has been reported in the cortex^8^ and diminished GABAergic perisomatic nets around neurons near amyloid plaques in APP/PS1 mice^36^. Further, a recent study revealed that the soluble APP fragment resulting from either the α-cleavage (sAPPα) or after the β-cleavage (sAPPβ) acts as an agonist on the GABA_B_ receptor^37^. The levels of sAPPs are expected to be elevated in APPswe/PS1dE9 mice since they have a 2.5-fold overexpression of human APP^38^. Therefore, we aimed to increase GABA_B_ tone in our APP/PS1 mice with a selective GABA_B_ receptor antagonist CGP-35348 to suppress SWDs. The selected dose was earlier shown to be effective in reducing SWDs in aged Wistar rats^39^. Nevertheless, we found no reduction in the occurrence of SWDs by this treatment. This finding can be interpreted in two ways. One interpretation suggests that the triggering mechanism for SWDs differs between aged APP/PS1 transgenic mice and aged rats, at least as far as the contribution of GABA_B_ receptors is concerned. The alternative interpretation is that due to the overexpression sAPPβ and chronic GABA_B_ agonist in APP transgenic mice they would require a larger dose of a GABA_B_ agonist than aged Wistar rats. Unfortunately, the present study design did not allow possibility to test more than one dose of each drug, but this possibility deserved dose–response assessment in future studies.

**Figure 7:**
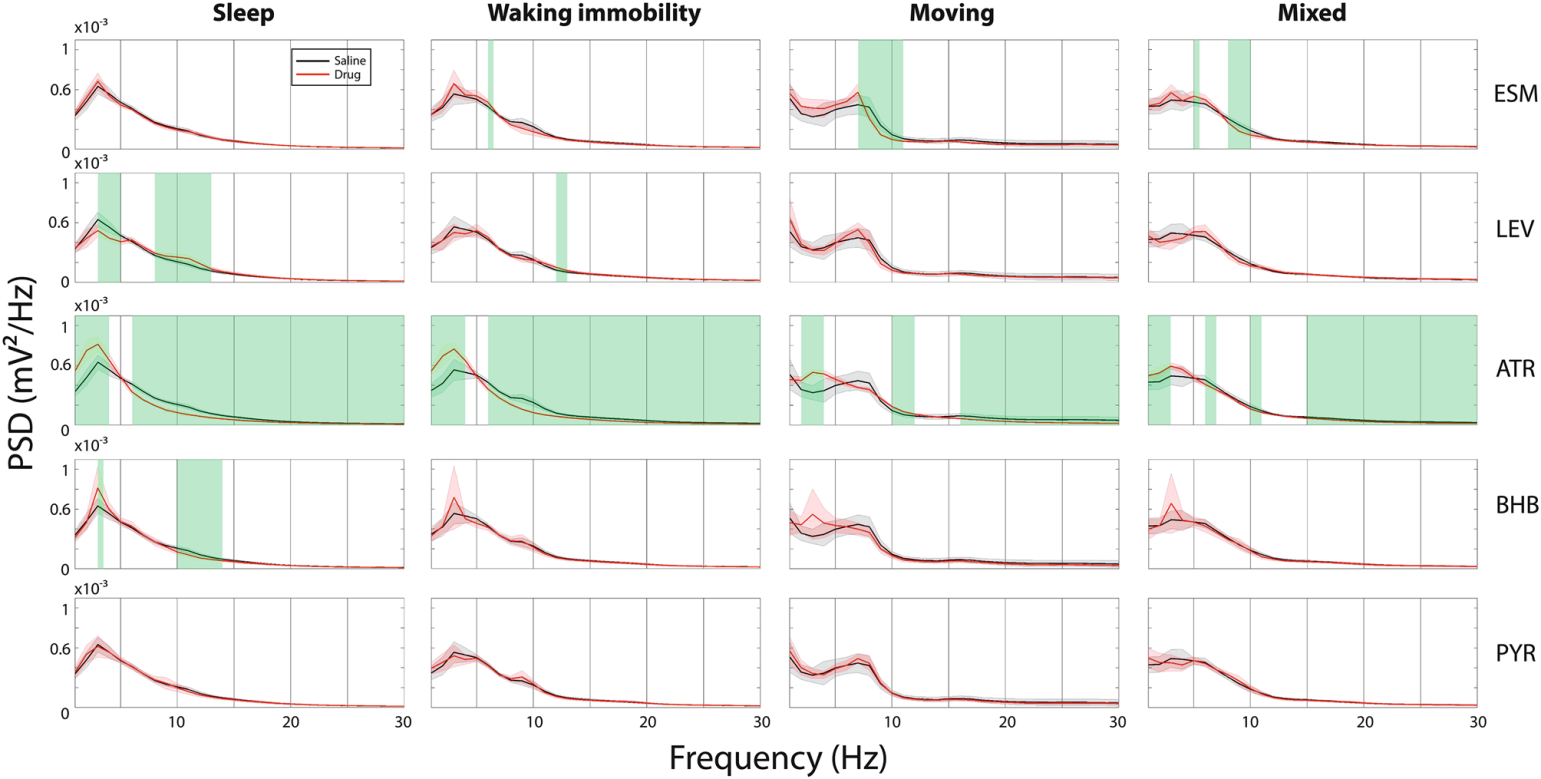
Comparison of relative mean PSD (± SD) within 1–30 Hz between effective drug administration and saline sessions in different behavioral states. ATR in all states increased relative delta power and decreased power at SWD ‘resonance’ frequency (9–11 Hz). ESM reduced power around 10 Hz in the mixed state and BHB in sleep, whereas LEV increased the power around 10 Hz in sleep. Green shading denotes frequency ranges where the drug session differed from the saline session at 0.05 level in FDR corrected independent samples t-test. All plots were binned by 1 Hz. For animal numbers see Table 1.

Reduced cholinergic tone due to degeneration of cholinergic nucleus basalis contributes to generation of high-voltage spindles, as SWDs used to be called in rats^11^. Tacrine, the first acetylcholinesterase inhibitor approved clinically for the treatment of AD, was shown to significantly suppress SWDs in aged Wistar rats at a dose of 3 mg/kg^40^, which also was most effective dose in improving spatial memory in aged rats^41^. Since APPswe/PS1dE9 mice display significant age-related attenuation of cholinergic neurotransmission in the cortex and hippocampus between 7 and 17 months of age^21^, we expected cholinesterase inhibitors to yield a similar effect in this specific AD model. We chose donepezil (DPZ) as a representative example of cholinesterase inhibitors currently in clinical use and adjusted the dose to 0.3 mg/kg based on the finding that 0.3 mg/kg but no more at 0.6 mg/kg improved learning and memory in APP transgenic mice^24^. Unexpectedly, we found no effect of DPZ at this dose on SWDs in aged APPswe/PS1dE9 mice. It is possible that a higher dose would have proved effective, since a recent study DPZ at the dose of 1 mg/kg significantly reduced the occurrence of SWDs in 12-month-old transgenic Fisher rats carrying human APPswe and PS1dE9 transgenes^42^. On the other hand, ACh or the synthetic nonselective cholinergic agonist carbachol increases excitability of rat cortical neurons, and the effect is blocked by atropine^43,44^. Thus, it would be expected that increased muscarinic ACh neurotransmission could also provoke SWDs. Indeed, we found a dramatic reduction in the SWD number by ATR that would have been expected to produce opposite effects based on the data on aged rats. Further, a recent study on APPswe transgenic mice reported that ATR at the standard dose of 50 mg/kg reduced the occurrence of interictal spikes in this mouse model^45^, which suggests that muscarinic receptor blockade rather than increased muscarinic cholinergic tone may reduce neuronal excitability in APP transgenic mice. However, these findings need to be interpreted with care. In the study of Kam and coworkers, interictal spikes were particularly frequent during REM sleep while ATR completely blocked REM sleep and increased locomotor activity^45^. We cut the ATR dose to a half but still failed to avoid general locomotor activation. However, this cannot be a confound in the present study, since we normalized the occurrence of SWDs (which are typically recorded during waking immobility or light sleep) to the time spent in waking immobility, sleep or mixed states. Moreover, ATR was the only treatment that changed the main frequency composition of SWDs by pushing their dominant frequency away from the ~ 9 Hz ‘resonance’ frequency. It also induced overall changes in the EEG power spectral density, especially increased delta power in all states, which resulted in decreased relative powers around the SWD ‘resonance’ frequency (Fig. 7). Together, these findings indicate that ATR at the same time decreased cortical excitability and slowed the cortical oscillation beyond the critical frequency for SWD occurrence. On the other hand, DPZ was reported to increase slow theta (4–6.5 Hz) and slow gamma (32–48 Hz) EEG power in Sprague Dawley rats during waking immobility at the dose of 1 mg/kg but not yet at 0.3 mg/kg^46^. It is possible that at a higher dose DPZ would have also reduced SWD occurrence but shifting the EEG power away from the ~ 9 Hz ‘resonance’ frequency.

Ketogenic diet has been proven to be effective, and sometimes the only treatment, in many childhood epilepsies, including absence epilepsy^14^. Our previous data suggest that chronic administration of the most important ketone substance beta-hydroxybutyrate (BHB) in combination with pyruvate reduces the occurrence of SWDs (in the paper called epileptiform discharges) in APPswe/PS1dE9 mice^15^. Further, chronic oral administration of pyruvate alone at ~ 450 mg/kg/day dramatically reduced the occurrence of epileptic spiking in rat models of focal and generalized convulsive seizures, as well as their occurrence in 6-month-old APPswe/PS1dE9 mice^47^. Notably, however, the latter study did not ascertain the effect of chronic pyruvate on SWDs. Collectively, these studies make alternate energy substrates beta-hydroxybutyrate (BHB), pyruvate (PYR) and lactate (LAC) an attractive treatment option for treating epileptic activity in AD patients, since both BHB^16^ and PYR^48^ have also shown neuroprotective effects in preclinical models of brain ischemia. However, the antiepileptic mechanisms of action of BHB, PYR and its breakdown product LAC have remained elusive. Besides providing alternate energy substrates to glucose, recent evidence suggests that BHB and PYR may have direct receptor mediated effects in the brain. BHB has been shown to specifically bind to hydroxycarbolic acid (HCA) 2 receptor in adipocytes, monocytes, macrophages and microglia, thereby altering lipolysis and the inflammatory response^16^. HCA 2 receptors are located also in the brain^16^ suggesting that BHB may exert its effect on brain excitability in a receptor-mediated action in addition to its metabolic action. Systemically administered PYR converts rapidly to LAC in the periphery but also reaches measurable levels in the brain when administered at large doses^17,18^. There is recent evidence that lactate binds specifically to G-protein coupled HCA 1 receptors, located in excitatory synapses in hippocampus, cerebellum and neocortex, and may thus influence neuronal excitability through this mechanism besides acting as an energy substrate^19^. We assumed that putative direct receptor-mediated effects of BHB, and LAC in particular, on excitatory neurotransmission would appear soon after an acute dose, while the inflammation modulating and metabolic effects may require repeated dosing.

The effect of the alternate energy substrates on SWDs was less robust but consistent. BHB tended to either decrease the number of SWDs or have no effect depending on the behavioral state, while PYR tended to increase SWDs occurrence at every behavioral state and LAC during waking immobility and mixed state. Notably, besides reduced relative power between 10 and 12 Hz by BHB during sleep, none of these substrates altered the frequency composition of SWDs or altered the EEG absolute power spectral density, suggesting that their effects were very different from those of atropine. Further, the opposite responses to BHB and PYR in the present study suggest that the SWD suppressing effect of the chronic BHB/PYR combination may arise primary from the BHB component^15^. However, these two studies cannot be directly compared, since administration of BHB or PYR in the chow resulted in slow fluctuations in the circulating levels and a total dose per day < 500 mg/kg, while the present study utilized acute injections of 1 g/kg of each. The literature is discrepant when it comes to brain concentrations of BHB, PYR or LAC after an i.p. injection. One microdialysis study in mice could detect only increase in brain glucose independent of whether the injected (2 g/kg) substance was BHB, PYR or LAC, while both LAC and PYR resulted in robust increase in blood lactate levels^18^. In contrast, another microdialysis study in rats demonstrated sustained increase brain levels of PYR or LAC after a 1 g/kg peripheral injection^17^. One careful interpretation of these two pharmacokinetic studies is that the chronically administered PYR most likely was metabolized to LAC already in the periphery and resulted in its use as an alternate energy substrate to glucose (resulting in increased extracellular glucose levels). In contrast, acute PYR was probably metabolized to LAC also in the brain. The present finding that acute LAC and PYR has practically identical effects on SWDs speaks for this alternative. With a high peak concentration, acute PYR and LAC may have induced direct HCA1 mediated effects, not seen after chronic administration. In that case, the SWD augmenting effect of PYR and LAC may have arisen through a HCA1 mediated action. Alternatively, the acute rise of LAC concentration in the brain may have resulted in cellular acidosis, which resulted in increased neuronal excitability. The BHB effect may have partially resulted from its binding to HCA2 receptors in brain resident macrophages and microglia. Such an immunomodulatory effect would be expected to be a slow response not reaching its peak effect during a couple of hours after an acute administration but well visible after 2–3 months of chronic intake. This would explain a more robust effect of BHB (in combination with PYR) on SWDs after chronic administration^14^.

The final important question is the translational value of SWDs in aged APP/PS1 mice in predicting drug effects on nonconvulsive epileptiform activity of MCI/AD patients. The relationship between spike-wave discharges in genetic rodent models and humans has been debated for decades^49^. On the one hand, their electrographic features are very different (e.g. main frequency 7–9 Hz vs. 3 Hz), and the rodent SWDs resemble more the human µ-rhythm seen around the central sulcus before and after movement^50^ and in certain types of epilepsies, such as progressive myoclonus epilepsy^51^ than human SWDs. On the other hand, rodent SWDs, human SWDs and human mu-rhythm all engage a similar thalamo-cortical loop^49^. No EEG study on AD patients to our knowledge has discovered SWDs, but so far only a couple of studies have more in detail described examples of any kind of nonconvulsive epileptic spiking in AD patients^2,52^. In contrast, two independent research groups have found substantial overrepresentation of SWDs in APP/PS1 mice compared wild-type littermates while the number of single cortical spikes did not differ between the genotypes^19,53^ and a recent study reported an eightfold higher occurrence of SWDs in APP/PS1 transgenic rats than in wild-type littermates^54^. Together, these findings suggest that SWDs are closely linked to brain amyloidosis. As such, they may serve as a surrogate marker for nonconvulsive spiking based on dysfunctional thalamo-cortical circuit even though a similar electrographic phenomenon will never be found in AD patients. Naturally, the most important criteria for the translational value of SWDs in AD mouse models to will be their predictive validity, i.e. whether drugs suppressing SWDs in APP transgenic mice will also suppress nonconvulsive spiking in MCI/AD patients. However, since no published data exist on the effect of various drug treatments on nonconvulsive epileptic activity in MCI/AD patients so far, the predictive validity of SWDs in APP/PS1 mice remains to be seen only in future clinical studies.

## Conclusion

The present study provides novel evidence that SWD responses to pharmacological treatment in amyloid plaque producing AD model mice is highly behavioral state dependent. This makes dose–response comparison between studies very difficult unless behavioral states are monitored and defined the same way. We found evidence that four kinds of drugs reduced SWDs at least at one behavioral state. ATR unexpectedly had the most robust effect that can be largely attributed to its effect on the network oscillations. However, this finding has little clinical relevance, since AD patients already display similar EEG slowing as induced by ATR^55^, but yields important mechanism insight into the mechanisms of SWD generation in APP/PS1 mice. All treatments that reduced the number of SWDs in a given state, also reduced either the absolute or the relative cortical EEG power around the SWD dominant frequency of ~ 9 Hz. ESM and LEV reduced SWD occurrence significantly during the mixed state, but probably through different mechanisms, since ESM decreased total cortical EEG PSD at SWD resonance frequency (9–11 Hz) while LEV did not change total cortical EEG PSD. This finding confirms earlier observations of their efficacy against SWDs in rat or mouse disease models. The finding that BHB reduced the occurrence of SWDs even after acute administration is consistent with the data derived from ketogenic diet and may prove significant also in the treatment of AD-related nonconvulsive epileptic activity.

### Ethics approval and consent to participate

The mice were kept in a controlled environment with food and water available ad libitum. All animal procedures were carried out in accordance with the guidelines of the European Community Council Directives 86/609/EEC and approved by the Animal Experiment Board of Finland.
